# An Efficient Synthesis of Enantiopure (*R*)-heteroarylpyrimidine Analogs

**DOI:** 10.3390/molecules180911144

**Published:** 2013-09-11

**Authors:** Xiu-Yan Yang, Guang-Qiang Xia, Xiao-Kui Wang, Zhi-Bing Zheng, Dong-Mei Zhao, Guo-Ming Zhao, Song Li

**Affiliations:** 1 School of Pharmaceutical Engineering, Shenyang Pharmaceutical University, Shenyang 110016, China; 2 Department of Medicinal Chemistry, Beijing Institute of Pharmacology and Toxicology, Beijing 100850, China

**Keywords:** anti-HBV, enantiopure, (*R*)-heteroarylpyrimidines, chiral separation, diastereomer

## Abstract

An efficient synthesis of enantiopure (*R*)-heteroarylpyrimidine analogs is described here, which involves introduction of a chiral group, formation and separation of diasteroisomers and final transformation of an amide to an ester. The absolute configuration of the enantiopure HAPs is confirmed by X-ray analysis of their intermediates.

## 1. Introduction

**Figure 1:**
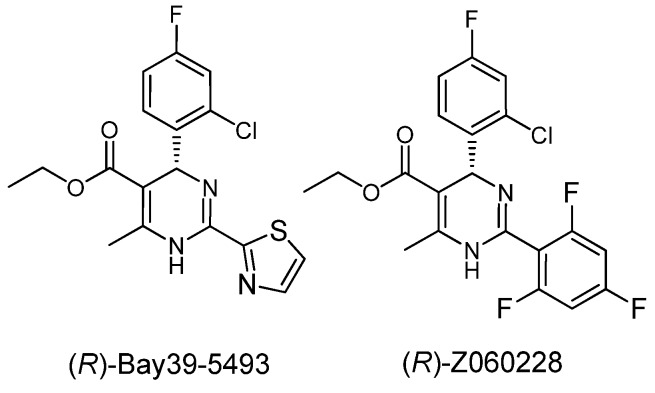
Structures of HAP analogs.

Chronic HBV infections remain a serious public health problem worldwide. Nucleoside/nucleotide analogs and immune modulators have been approved for the treatment of chronic hepatitis B. Unfortunately, drug resistance and side effects have limited the utility of currently approved drugs [1,2]. Therefore new kinds of anti-hepatitis B agents are still highly desired. Heteroarylpyrimidines (HAPs) were discovered to be a class of highly potent non-nucleoside inhibitors of HBV replication (Figure 1) [3,4,5]. Bay39-5493 has reached the clinical test stage as an anti-HBV candidate drug. **Z060228**, another novel HAP derivative found by our laboratory, exhibits excellent activity against HBV replication at submicromolar concentration and is currently under preclinical study [6,7].

**Scheme 1:**
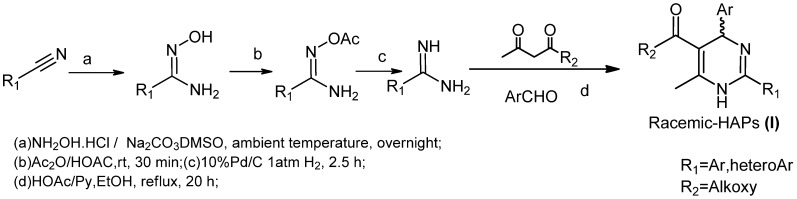
Synthesis of racemic HAPs.

The biological activity of HAPs depends on their absolute configuration and only the (*R*)-enantiomers exhibit anti-HBV activity, so the synthesis of (*R*)-HAPs is necessary for the further drug development. Racemic HAPs were easily prepared from amidine, ethyl acetoacetate and benzaldehyde by a Biginelli reaction (Scheme 1) [8]. However, enantiopure (*R*)-HAPs were difficult to obtain and only Goldman *et al.* have reported the preparation of (*R*)-Bay39-5493 through a chiral-phase HPLC method [9,10]. Herein, we report on a feasible and convenient synthesis of enantiopure (*R*)-HAPs.

## 2. Results and Discussion

**Scheme 2:**
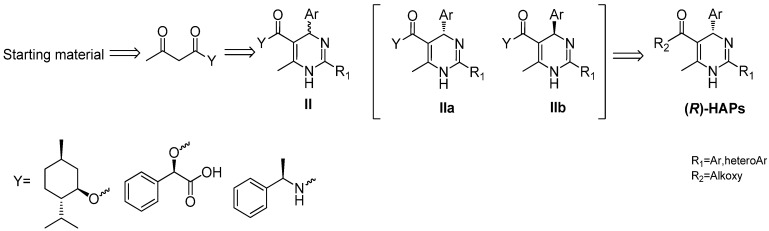
Synthetic strategy analysis of enantiopure (*R*)-HAPs.

In general, the methods frequently used for the synthesis of enantiopure 1,4-dihydropyridines are the resolution of racemic dihydropyridines, separation via diastereomeric esters, enantioselective synthesis with chiral auxiliary groups, chemoenzymatic separation of dihydropyridines, and chromatographic separation of enantiomers [11,12,13]. The synthetic strategies we first adopted for the preparation of (*R*)-HAPs involved resolution of the racemic-HAPs I via diastereomeric salts using camphorsulfonic acid as resolution reagent and direct enantioselective synthesis in the chiral environment of quinidine or quinine, but the results were not satisfactory. Then we attempted an indirect method with a chiral auxiliary group to synthesize (*R*)-HAPs as shown in Scheme 2. A chiral group **Y** was introduced in starting material and a couple of diasteroisomers **II** were formed by the Biginelli reaction. The enantiopure **IIa** and **IIb** could be separated by taking advantage of the differences in their physiochemical properties and then the enantiopure **IIa** could be transformed into (*R*)-HAPs after getting rid of the introduced chiral group **Y**. Apparently, the choice of chiral group **Y** is key for the whole strategy. According to their cost and practical properties, several chiral agents such as menthol, and mandelic acid were adopted. When menthol was used, the enantiopure **IIa** or **IIb** were not crystallized easily from common solvents. When mandelic acid was used, racemization was found to occur under basic conditions. By comparison, (*R*)-1-phenylethanamine was proved to be the suitable chiral agent, which was enantiomerically stable in the subsequent reaction steps and was removed conveniently after enantiopure intermediate **IIa** was separated.

**Scheme 3:**
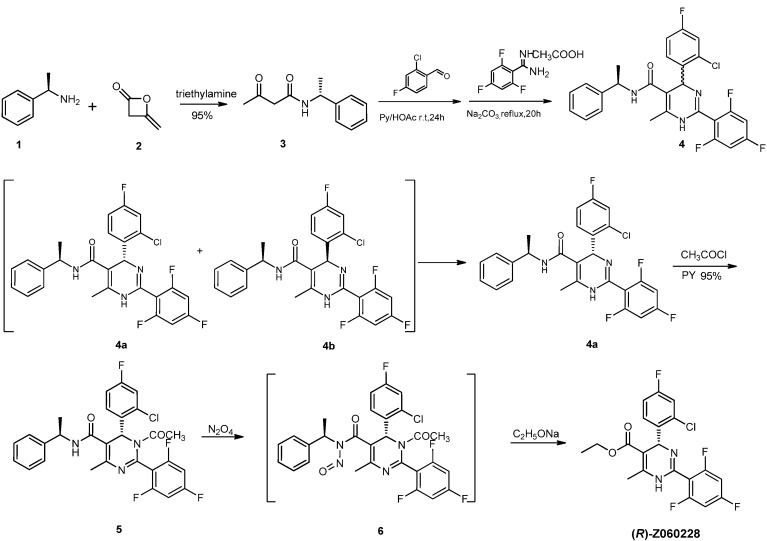
Synthesis of **(*R*)-Z060228**.

With this method **(*R*)-Z060228** has been synthesized by the synthetic route shown in Scheme 3. In the first step, acylation of (*R*)-1-phenylethanamine (**1**, >99% ee) with diketene (**2**) easily gave the intermediate **3** in 95% yield [14]. Next, Knoevenagel condensation of **3** with 2-chloro-4-fluorobenzaldehyde in the presence of acetic acid and piperidine followed by reaction with 2,4,6-trifluorobenzimidamide acetate afforded the diastereomeric **4** in 78% yield [15,16,17].

As we expected, the diastereomers **4a** and **4b** have different solubility in different solvents. Compound **4a** was easily crystallized from ethyl acetate, whose *de* value was >99% according to HPLC and then **4b** was easily recrystallized from ethanol.

Next we focused our attention on the transformation of amide **4a** into the ester (***R***)**-Z060228**, which was also a key step of the synthetic route. In general, esters aren’t easily obtained from the corresponding amides. Alcoholysis of amides, especially for polyfunctional amide was problematic because conventional methods under strongly basic and acidic conditions were only suitable for simple amides and otherwise result in extensive substrate decomposition [18]. What’s more, the amide **4a** has lower activity because of its 1,4-dihydropyrimidine ring. In order to improve the reactivity of **4a**, introduction of an electron-withdrawing group on the amide-*N* atom of **4a** was considered Methyl chloroformate and glutaric anhydride were firstly selected as activation reagents, but the results were not satisfactory because of **4a**’s steric effect. Fortunately, we found that *N*-nitrosamide formation was also an efficient method for activation of amides. Nitrosation of secondary amines is usually accomplished using nitrosating agents such as nitrous acid, NaNO_2_/HC1, nitrogen oxides (NO, N_2_O_3_ or N_2_O_4_) and so on. To improve the yield and avoid the formation of side products, dinitrogen tetroxide was considered as a selective and efficient reagent for N-nitrosation of the secondary amine **4a** [19,20,21,22,23,24,25].

**Scheme 4:**
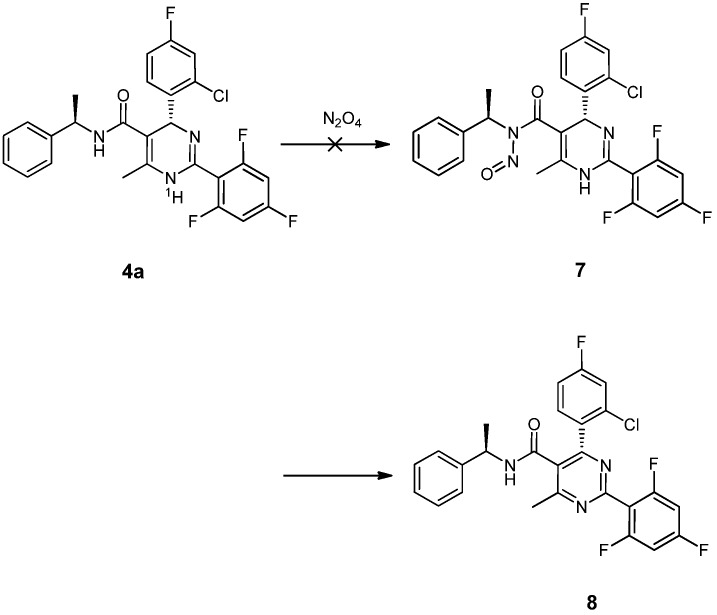
Direct *N*-nitrosation only yielded an arylate product.

However, in the course of our experiments, direct *N*-nitrosation of compound **4a** with dinitrogen tetroxide afforded the undesired product **8** in almost 100% yield instead of the desired compound **7**. It is noteworthy that this accidental discovery might actually be an excellent method to prepare substituted pyrimidines. According to the analysis of the reaction and the product we concluded that the higher activity of N^1^-H than the amide N-H may be the main cause (Scheme 4). To overcome this problem, protection of the amide N^1^-H was required. Acylation of **4a** with acetyl chloride in the presence of triethylamine gave the intermediate **5** in good 95% yield. *N*-Nitrosation of **5** with dinitrogen tetroxide easily gave the intermediate **6**. Then alcoholysis of crude **6** with sodium ethoxide in cold dry DMF afforded the desired compound **(*R*)-Z060228** in 78% yield.

**Figure 2:**
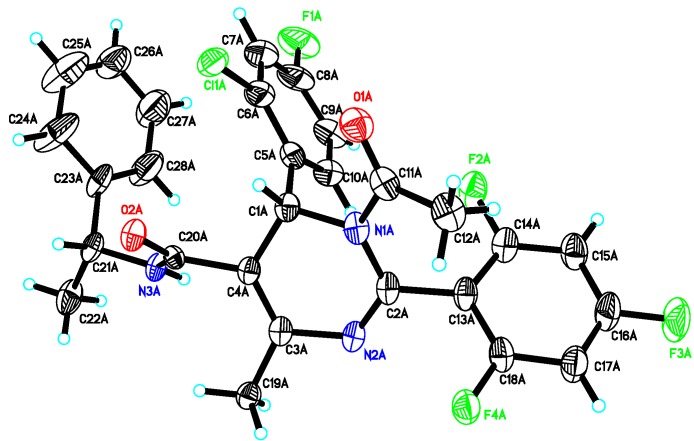
X-ray crystal structure of the intermediate **5** [28].

**(*R*)-Z060228**’s optical purity (>99% ee) was checked with HPLC on a chiral stationary phase (ULTRON ES-OVM 150 × 4.6 mm, 0.01M KH_2_PO_4_:CH_3_CN = 90:10, λ = 240 nm, 1.0 mL/min), which indicated that no racemization occurred in the last two steps. **(*R*)-Z060228**’s specific rotation was found to be 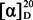 = −92.4 (c = 1.0, CH_3_OH) and its absolute (*R*) configuration could be directly confirmed by determination of the absolute configuration of the intermediate **(*4R,2'R*)-5** by X-ray crystallography (Figure 2) [26]. Using a similar reaction sequence, **Bay39-5493** was prepared in 99% ee and its specific rotation closely matched the reported value (observed: 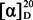 = −54.6, c = 1.0, CH_3_OH; lit.: 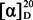 = −52, c = 1.0, CH_3_OH) [27].

## 3. Experimental

### 3.1. General

^1^H-NMR and ^13^C-NMR spectra were recorded at 400 MHz and 100 MHz on a JNM-ECA-400 instrument with tetramethylsilane as an internal standard in the DMSO. ESI-MS (high resolution) mass spectra were obtained by using a Waters Xevo G2 Qtof (ESI) mass spectrometer. Melting points were determined using a RY-1 apparatus and are uncorrected.

### 3.2. Synthesis

*R,R-N-(1-Phenylethyl)-4-(2-chloro-4-fluorophenyl)-2-(2,4,6-trifluorophenyl)-6-methyl-1,4-dihydro-pyrimidine-5-carboxamide* (**4a**) *and R,S-N-(1-phenylethyl)-4-(2-chloro-4-fluorophenyl)-2-(2,4,6-trifluorophenyl)-6-methyl-1,4-dihydropyrimidine-5-carboxamide* (**4b**). (*R*)-(+)-*N*-(1-phenylethyl)-3-oxobutyramide (**3**, 4.50 g, 22.05 mmol) and 2-chloro-4-fluorobenzaldehyde (3.50 g, 22.05 mmol) are dissolved in anhydrous alcohol (85 mL) containing acetic acid (1 mL) and piperidine (1 mL) as catalysts. After stirring at R.T. for 24 h, 2,4,6-trifluorobenzimidamide acetate (3.51 g, 22.05 mmol) and sodium acetate (2.05 g, 25.00 mmol) are added. After refluxing for 20 h, the reaction mixture is concentrated and the residue is dissolved in water (50 mL) and extracted with ethyl acetate (3 × 25 mL). The combined organic extracts are washed with brine (20 mL × 3) and dried over anhydrous sodium sulfate. After evaporation of the solvent *in vacuo*, a 1:1 mixture of diastereomeric amides **4a** and **4b** is obtained and subjected to recrystallization from ethyl acetate and pentane (50:50), **4a** (3.9 g, 35%) is isolated as white particles (>99% d.e., according to HPLC). R_f_ = 0.34 (ethyl acetate-petroleum ether = 2:1). Mp = 208–210 °C. ^1^H-NMR (DMSO-*d_6_*) δ: 1.29–1.33 (3H, m); 1.97 (3H, s); 4.83–4.87 (1H, m); 5.97 (1H, s); 7.04–7.06 (2H, d, *J* = 6.4 Hz); 7.12–7.24 (7H, m); 7.25–7.27 (1H, m); 8.08–8.10 (1H, d, *J* = 8 Hz); 9.24 (1H, s); HRMS (ESI) *m/z*: Calcd for C_26_H_21_ClF_4_N_3_O, 502.1304 [M+H]^+^; Found: 502.1299.

The mother liquor was concentrated and redissolved in anhydrous alcohol, whereby **4b** is crystallized (3.43 g, yield 31%) as white plates (>99% d.e., according to HPLC). R_f_ = 0.36 (ethyl acetate-petroleum ether = 2:1). Mp = 197–199 °C. ^1^H-NMR (DMSO-*d_6_*) δ: 1.17–1.25 (3H, m); 2.12 (3H, s); 4.85–4.88 (1H, m); 6.01 (1H, s); 7.15–7.20 (8H, m); 7.86–7.87 (2H, d, *J* = 4 Hz); 7.94–7.95 (1H, m); 9.37 (1H, s); HRMS (ESI) *m/z*: Calcd for C_26_H_20_N_3_OF_4_Cl, 502.1304 [M+H]^+^; Found: 502.1299.

*R,R-N-(1-phenylethyl)-N-1-acetyl-6-(2-chloro-4-fluorophenyl)-2-(2,4,6-trifluorophenyl)-4-methyl-1,6-dihydropyrimidine-5-carboxamide* (**5**). A solution of diastereomeric amide **4a** (3.03 g, 6 mmol) and anhydrous triethylamine (0.9 g, 9 mmol) in dry DMF (45 mL) is stirred at 0 °C, and then acetyl chloride (0.51 mL, 7.2 mmol) is added dropwise. After stirring for 6 h, the reaction mixture is quenched with water (60 mL), and extracted with ethyl acetate (3 × 60 mL). The combined organic phase is dried over anhydrous sodium sulfate and concentrated under reduced pressure. The residue is purified by column chromatography on silica gel (ethyl acetate-pentane = 1:3) to give **5** (3.11 g, 95%) as a white powder. Rf = 0.4 (ethyl acetate-petroleum ether = 1:1). Mp: 183–184.2 °C. ^1^H-NMR (DMSO-*d_6_*) δ: 1.39–1.41 (3H, d, *J* = 8 Hz), 2.07 (3H, s), 2.10 (3H, s), 4.95–4.99 (1H, m), 6.47 (1H, s), 7.08–7.12 (3H, m), 7.15–7.30 (5H, m); 7.45–7.51 (2H, m), 8.70–8.72 (1H, d, *J* = 8 Hz). HRMS (ESI) *m/z*: Calcd for C_28_H_23_N_3_O_2_ClF_4_, 544.1409 [M+H]^+^; Found: 544.1398.

*Ethyl 4-(R)-(2-chloro-4-fluorophenyl)-2-(2,4,6-trifluorophenyl)-6-methyl-1,4-dihydropyrimidine-5-carboxylate* **(R)-Z060228**. To a solution of **5** (2.71 g, 5 mmol) in dichloromethane (50 mL) at 0 °C is added dinitrogen tetroxide (4.60 g, 50 mmol, 10 eq.). The solution is stirred under nitrogen at 0 °C for 20h and then poured over ice, and extracted with cold dichloromethane (2 × 200 mL), the organic part is concentrated in vacuum in an ice water bath to yield a yellow oil, which is dissolved in cold dry DMF (150 mL, −40 °C), and sodium ethoxide (6.8 g, 100 mmol, 20 eq.) is added. The mixture is stirred for 15 min under nitrogen atmosphere and then quenched with water (200 mL) neutralized with 4 M HCl, and extracted with ethyl acetate (150 mL × 3). The combined extracts are dried over anhydrous sodium sulfate, and concentrated at reduced pressure. The residue is purified by column chromatography on silica gel (ethyl acetate-dichloromethane 4:100) to give **(*R*)-Z060228** (1.66 g, 78%) as a white powder. 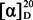 = −92.4 (c = 1.0, CH_3_OH), ^1^H-NMR (DMSO-*d_6_*) δ: 1.02–1.05 (3H, t); 2.32 (3H, s); 3.92–3.96 (2H, m); 5.97 (1H, s); 7.21–7.45 (5H, m); 9.86 (1H, s). ^13^C-NMR (DMSO-*d_6_*) δ: 14.4, 17.9, 56.0, 59.6, 96.2, 101.3, 101.5, 101.8, 115.2, 116.6, 116.8, 131.2, 131.2, 132.6, 139.8, 142.0, 147.6, 160.0, 162.4, 166.1. HRMS (ESI) *m/z*: Calcd for C_20_H_15_N_2_O_2_F_4_Cl, 427.0831 [M+H]^+^; Found: 427.0827.

*Ethyl 4-(R)-(2-chloro-4-fluorophenyl)-2-(thiazol-2-yl)-6-methyl-1,4-dihydropyrimidine-5-carboxylate* **(R)-Bay39-5493**. 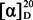 = −54.6 (c = 1.0, CH_3_OH). ^1^H-NMR (DMSO-*d_6_*) δ: 1.01–1.04 (3H, m); 2.50 (3H, s); 3.90–3.94 (2H, m); 5.99 (1H, s); 7.36–7.43 (3H, m); 7.90–7.91 (1H, d, *J* = 4 Hz); 7.97–7.98 (1H, d, *J* = 4 Hz); 9.96 (1H, s); HRMS (ESI) *m/z*: Calcd for C_17_H_15_N_3_O_2_FSCl, 380.0630 [M+H]^+^; Found: 380.0628.

## 4. Conclusions

In summary, a novel and efficient approach to the synthesis of enantiomerically pure HAPs is accomplished from inexpensive starting materials. Key feature of this synthesis include an introduction of another chiral group and the alcoholysis to yield the final product. The method described herein could be an attractive alternative for the synthesis of chiral HAPs.
